# Outcomes of LASIK for Myopia or Myopic Astigmatism Correction with the FS200 Femtosecond Laser and EX500 Excimer Laser Platform

**DOI:** 10.2174/1874364101812010063

**Published:** 2018-05-18

**Authors:** Muanploy Niparugs, Napaporn Tananuvat, Winai Chaidaroon, Chulaluck Tangmonkongvoragul, Somsanguan Ausayakhun

**Affiliations:** Chiang Mai University LASIK Center, Center for Medical Excellence, and Department of Ophthalmology, Faculty of Medicine, Chiang Mai University, Chiang Mai, Thailand

**Keywords:** Laser *in situ* Keratomileusis (LASIK), Myopic correction, Myopic astigmatism, Femtosecond laser, Excimer laser, UDVA

## Abstract

**Purpose:**

To evaluate the efficacy, predictability, stability and safety of laser in situ keratomileusis (LASIK) using the FS200 femtosecond laser and EX500 excimer laser platform.

**Methods:**

The outcomes of 254 eyes of 129 consecutive patients with myopia or myopic astigmatism who underwent full correction femtosecond laser-assisted LASIK at CMU LASIK Center were assessed. Pre-operative and post-operative parameters including manifest refraction, Uncorrected Distance Visual Acuity (UDVA), Best Corrected Distance Visual Acuity (BDVA), corneal topography and tomography were analyzed. The results between low to moderate myopia and high myopia were compared up to 12 months.

**Results:**

Mean pre-operative Spherical Equivalent (SE) was -5.15±2.41 Diopters (D) (range -0.50 to -11.50 D) and -0.13±0.28 D, -0.13±0.27 D, -0.13±0.28 D and -0.14±0.30 D at 1, 3, 6, and 12 months, post-operatively. At 12 months, the propor¬tion of eyes achieving UDVA ≥ 20/20 was 90.0% and ≥20/40 was 98.8%. The proportion of eyes achieving post-operative mean SE ±0.5 D, and ±1 D was 91.3%, and 98.5%. No eyes lost more than two lines of BDVA. The low to moderate myopic group had a statistically significant better UDVA at one (*p*=0.017) and three months (*p*=0.014) but no difference at six (*p*=0.061) and 12 months (*p*=0.091). The mean post-operative SE was better in low to moderate myopic group at every follow-up visit (*p*=0.001, 0.007, <0.001 and <0.001).

**Conclusion:**

One-year clinical results of LASIK with the FS200 femtosecond laser and EX500 excimer laser showed high efficacy, predictability, stability and safety.

## 1 INTRODUCTION

Currently, corneal laser refractive surgery is the most used method to correct various types and degrees of refractiv errors. The improvement of technologies and innovations continues at a rapid pace. New laser platforms have been developed to increase the accuracy and safety of the procedures.

In the procedure of laser in situ keratomileusis (LASIK), the excimer laser is used during the second step after the flap creation to correct refractive error. Excimer ablation has significantly improved over the past ten years with the introduction of improvement in energy delivery to the cornea and of new ablation algorithms in clinical practice [1].

For LASIK flap creation, the femtosecond laser has become a rapidly and widely adopted technology to enhance the precision, reproducibility, safety, and clinical outcomes of the procedure. Femtosecond created flaps were found to deliver more accurate, reproducible flaps with uniform thickness and reduced incidence of flap complications compared to microkeratome created flaps [2-6].

The Wavelight refractive suite (Alcon Laboratories Inc, USA), introduced in 2010, is the integration of the femtosecond laser and the excimer laser into one system, connected by the WaveNet integrated computer network. The FS200 femtosecond laser delivers laser of 200 KHz frequency which increases the speed of flap creation and provides precise and predictable outcomes. It combines a small focus with low pulse energy and a unique cutting pattern for accurate flap creation [4]. The EX500, 500 Hz repetition rate, delivers faster excimer laser and its innovative engineering helps reduce the potential for sensitivity to eye movements, patient eye fixation fatigue, flap shrinkage and stromal dehydration of the cornea caused by delayed or prolonged treatment times [7]. This excimer laser operates with a high pulse repetition with the 1,050-Hz multidirectional eye tracker, providing precise ablation quality and shorter treatment time. These advantages result in consistent outcomes [1, 8].

However, there are few published studies regarding the clinical outcomes of refractive error correction by femtosecond laser-assisted LASIK (F-LASIK) with FS200 femtosecond laser and EX500 excimer laser platform [9-12].

The purpose of this study was to evaluate the efficacy, predictability, stability and safety in myopic and myopic astigmatism corrective LASIK procedure when using the FS200 femtosecond laser and the EX500 excimer laser platform. The results from the low to moderate myopic group and high myopic group were also compared.

## 2 MATERIALS AND METHODS

In accordance with the principle of the Declaration of Helsinki, this study was approved by the Research and Ethics Committee, Faculty of Medicine, Chiang Mai University before initiated. This study enrolled consecutive patients with myopia or myopic astigmatism who underwent full correction LASIK procedure by using the FS200 femtosecond laser and EX500 excimer laser platform (WaveLight^®^; Alcon Laboratories Inc., USA) with Wavefront optimized profile from January to December 2013 at the Chiang Mai University LASIK Center.

Exclusion criteria for the LASIK surgery included patients with coexisting ocular or systemic disease, unstable refractive error, manifest or suspected corneal ectatic disorders, eye with history of herpetic keratitis or corneal dystrophy, corneal scarring, cataract, glaucoma, uncontrolled diabetes, collagen vascular disease, pregnancy, and in patients taking isotretinoin or hormonal therapy.

Patients were also excluded from this study if their age was less than18 years, post-operative follow-up time of less than 12 months, and pre-operative Best corrected Distance Visual Acuity (BDVA) of less than 20/20.

Visual acuity, manifest and cycloplegic refraction were performed using the Early Treatment of Diabetic Retinopathy (ETDRS) chart at 4 meters under photopic lighting condition. All patients were also evaluated by corneal topography (WaveLight^®^Topolyzer™ VARIO Diagnostic Device; Alcon Laboratories), corneal tomography (WaveLight®Oculyzer II; Alcon Laboratories), and scotopic pupillometry (Colvard pupillometer). A complete eye examination was performed which included applanation tonometry, slit-lamp biomicroscopy and dilated fundus examination. Pre-operative dry eye and retinal problems were treated prior to surgery.

All surgeries were performed by four surgeons with similar techniques. FS200 laser pulse energy and scanning parameters were the same for all treatments. The pulse energy was set at 0.8 μJ, and the spot and line separation for the bed cut was 8 μm, while a spot separation of 5 μm and line separation of 3 μm were used for a sidecut. The FS200 laser was programmed for each procedure with a flap thickness of 100-120 μm, the side cut angle of 70 degrees, and superior hinge with hinge width of 0.4 mm. All eyes had estimated Residual Stromal Bed Thickness (RSBT) more than 270 µm (mean 349.61±38.61µm, range 272 to 444). For EX500 laser, all eyes underwent wavefront optimized ablation and the laser setting utilized the WaveLight’s company-supplied normogram. Post-operative medications were a combination of topical steroids with antibiotics (Vigadexa^®^; Alcon Laboratories) four times per day for one week and frequently administered non-preserved lubricants.

At each postoperative follow-up visit, all patients were asked to grade the symptoms of dry eye and night vision difficulties in each eye with the score of zero to five (from none to the maximum symptoms). All eyes were evaluated for uncorrected distance visual acuity (UDVA), BDVA, manifested refraction, corneal tomography, intraocular pressure, and anterior segment examination at one month, three months, six months, and 12 months. A dilated fundus examination was performed postoperatively at six and 12 months. The refractive results of the low to moderate myopic group (manifest refractive spherical equivalent, MRSE < 6 Diopters, D) were compared to the high myopic group (MRSE ≥ 6 D).

Data were analyzed using SPSS version 17.0 (SPSS Inc., Chicago, Illinois). All proportions were presented as number and percentage. The data collected on the continuous or ordinal scale were expressed as mean, minimum, and maximum. Chi-square tests were used to compare the proportion data of the two groups and independent samples t-tests were used to compare mean data of the groups. A p-value <0.05 was considered as statistically significant.

## 3 RESULTS

### 3.1 Patients and Pre-operative Parameters

A total of 254 eyes from 129 patients were recruited. Forty-four patients (34.1%) were males and 85 patients (65.9%) were females, with a mean age of 31.54± 8.61 years (range 18-64). One hundred twenty-six left eyes (49.6%) and 128 (50.4%) right eyes were included.

The mean pre-operative UDVA (LogMAR) was 1.05±0.31 (0.1 to 1.6). The mean MRSE was -5.15±2.41 D. The mean sphere was -4.74±2.37 D (0 to -11.0) and the mean cylinder was -0.83 ± 0.79 D (0 to -3.75). Other pre-operative parameters were the following; the mean keratometry was 44.06±1.27 D, the mean central corneal thickness was 543.77±27.65 µm, the mean white to white was 11.71±0.32 mm and the mean scotopic pupil size was 6.06±0.62 mm.

### 3.2 Post-operative Refractive Results

**Fig. (2):**
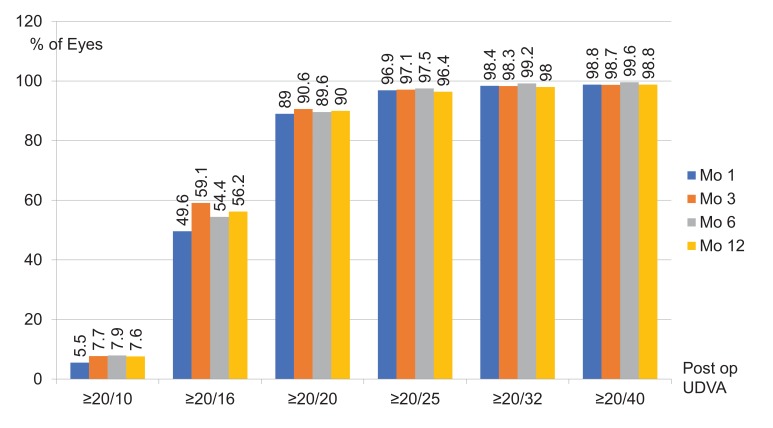
Post-operative uncorrected distance visual acuity at 1, 3, 6, and 12 months after LASIK.

**Fig. (3):**
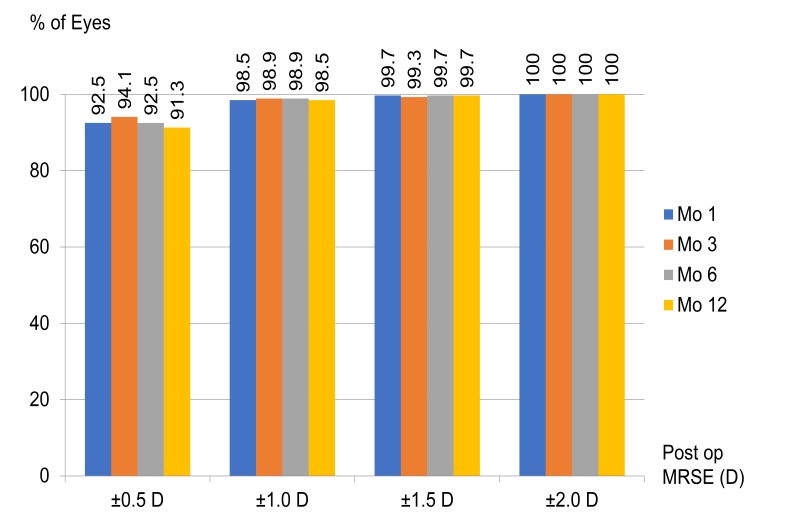
Post-operative mean spherical equivalent refraction at 1, 3, 6, and 12 months .

After LASIK, the mean MRSE was -0.13±0.28 D, -0.13±0.27 D, -0.13±0.28 D and -0.14±0.30 D at 1, 3, 6, and, 12 months, respectively. The refractive changes are demonstrated in Fig. (**1**). The proportion of eyes achieving post-operative UDVA ≥20/20 was ≥ 89.0% and ≥20/40 was ≥98.7% in 12 months (Fig. **2**). The proportion of eyes achieving post-operative mean MRSE lower than 0.5 D was ≥91.3% and lower than 1.0 D was ≥98.5% in 12 months (Fig. **3**).

Fig. (**4**) demonstrates the change of BDVA after surgery, most of the eyes were unchanged or gained one line of BDVA (about 80 to 85% of the eyes). At one year, 58.8% had unchanged BDVA, 27.2% gained one line, 14.0% lost one line and no eyes lost more than one line.

For subgroup analysis, the refractive results of the low to moderate and the high myopic group were compared. One hundred and sixty-one eyes (63.4%) were in the low to moderate group and ninety-three eyes (36.6%) were in the high myopic group. The mean MRSE was -3.60±1.34 D in the low to moderate and -7.83±1.18 D in the high myopic group. There were no statistically significant differences in the demographic data between groups except preoperative mean SE and UDVA (Table **1**).

When comparing post-operative refractive outcomes between groups, the proportion of eyes achieving better UDVA was shown to be statistically significantly greater in the low to moderate myopic group at one (*p*=0.017) and three months (*p*=0.014) but had no significant differences at six (*p*=0.061) and 12 months (*p*=0.091). The low to moderate myopic group had a better mean post-operative MRSE than the high myopic group at every follow-up visit (*p*=0.001, 0.007, <0.001 and <0.001). The change of BDVA after surgery was not different between groups (Table **2**).

### 3.3 Intra- and Post-operative complications

Intra-operative complications were documented in 24 eyes (9.5%), including bleeding under the flap in ten eyes (3.94%) and among these, two eyes required flap irrigation; peripheral Opaque Bubble Layer (OBL) in six eyes (2.36%) and in each case the surgery continue without any further flap complications, such as a tear or a button hole, in all eyes. An air bubble occurred in the anterior chamber of three eyes (1.18%) and flap displacement was noted immediately post-surgery in three eyes (1.2%) with epithelial abrasion in two eyes (0.79%).

During the post-operative follow-up visits, a mild degree of Diffuse Lamellar Keratitis (DLK) was found in ten eyes (3.94%), all occurred within the first week after surgery and resolved with topical corticosteroid. No records of significant epithelial ingrowth and symptoms of rainbow glare or transient light sensitivity syndrome were reported. Post-operative dry eye signs and symptoms, and night vision problems were demonstrated in Table **3**.

The average scores of dry eye symptoms and night vision problems after LASIK were less than two and decreased with time after surgery. Symptoms of dry eye were correlated with corneal staining which improved with time after surgery (Table **3**).

One eye (0.39%) developed rhegmatogenous retinal detachment (RRD) at one month after LASIK. This patient had pre-operative MRSE of -8.38 D and underwent prophylactic focal laser retinopexy for retinal break before LASIK procedure. However, a new retinal break with localized RRD that spared the macula was found. He eventually underwent retinal surgery and his final UDVA was improved up to 20/20. One eye (0.39%) had a new retinal break without retinal detachment on routine fundus examination at the six-month post-LASIK exam.

LASIK enhancement was performed in four eyes (1.57%), two eyes at three months and the other two eyes at eight months after primary LASIK procedures. All eyes gained UDVA of 20/20 after the enhancement. No eyes developed signs of corneal ectasia at one year post surgery.

## 4 DISCUSSION

The technology of the femtosecond laser reached 500 kHz from its original 6 kHz. The higher laser frequency permits lower energy per pulse, shorter flap-cutting time and tighter spot separation, which leads to smoother corneal stromal bed creation resulting in better visual and refractive outcomes after LASIK [2, 13].

The safety and success of LASIK are related to consistent and precise flap creation. Previous studies found that flap creation with the FS200 femtosecond laser appeared to be safe and predictable with high accuracy. By using the Anterior Segment Optical Coherence Tomography (ASOCT), Kymionis et al. found that the achieved mean central flap thickness deviated less than 3 μm (102.98μm) from the attempted thickness (105 μm) and showed small variability (SD 6.33 μm) at one month post-operation [10].

Cummings et al. also showed the predictability and lower variability of LASIK flaps created by FS200 laser using online pachymetry and ASOCT. With an intended flap thickness of 120 μm, the mean post-LASIK flap thickness was 120.23μm ± 19.94 (SD). In addition, achieved flap dimensions were as attempted [11].

One systematic review and meta-analysis study evaluated the visual and refractive outcomes of F-LASIK using various femtosecond lasers including IntraLase FS10, 15, 30, 60 and 150 kHz, FEMTO LDV, Visumax, and Wavelight FS200 found that the FS200 had satisfactory outcomes in terms of safety, predictability, and stability similar to other femtosecond lasers. In addition, LASIK flap creation with FS200 had the fewest intra-operative and post-operative complications compared to the other lasers [14].

Cummings et al. studied the outcomes of myopic F-LASIK correction using the FS200 and EX500 lasers and found that 83.4% of eyes had UDVA of ≥ 20/20, 91.0% had post-operative MRSE lower than 0.5 D. The percentage of patients who had unchanged CDVA was 69%, gained one line was 20.2% at three months postoperatively [11]. Recently, a prospective multicenter study on the outcomes of 96 patients with myopia and myopia with astigmatism correction with F-LASIK using the Wavelight® refractive suit found that postoperative UDVA was significantly superior to preoperative CDVA. (Mean binocular UDVAs at 1, 3, and 6 months were -0.088 ± 0.107, -0.098 ± 0.107, and -0.105 ± 0.113 logMAR compared with mean preoperative binocular CDVA of -0.025 ± 0.126 logMAR) [12].

Kanellopoulos et al. reported the one year results of 190 eyes that underwent myopic F-LASIK using the FS200 femtosecond and EX500 excimer laser and found that 94.7% of eyes had postoperative UCVA ≥20/20 at month three, and maintained this until month 12. The proportions of eyes achieving postoperative MRSE lower than 0.5 D were more than 87% and 95.7% were lower than 1.0 D at 12 months [9]. When compared to this study, their results seem to have slightly better results, which may be because our study had a wider range of pre-operative MRSE (-0.50 to -11.50 D) compared to those in their study (-0.50 to -8.00 D) as well as a greater proportion of eyes had higher degree of myopia.

For the results from eyes with different degree of myopia, the low to moderate myopic group had a statistically significantly greater proportion of eyes achieving better UDVA compared with the high myopic group at short-term follow-up post-surgery. However, the long-term refractive results were similar in both groups. Nonetheless, the low to moderate myopic group mean post-operative MRSE was better than that of the high myopic group at every follow-up visit, the average mean post-operative MRSE of both groups was less than ±0.5 D.

The visual and refractive outcomes of F-LASIK demonstrated excellent safety and efficacy, with most studies reporting equivalence with microkeratome LASIK [13, 15]. Femtosecond created flaps were found to deliver more accurate, reproducible flaps with uniform thickness and reduced incidence of flap complications [2, 4-6]. However, there are some complications specific to femtosecond lasers such as Opaque Bubble Layer (OBL), intracameral bubbles, Transient Light Sensitivity Syndrome (TLSS), rainbow glare and higher incidence of DLK [5]. However, the reduced energy requirements in the new generation of femtosecond laser is supposed to reduce the cavitation bubble size and duration, tissue inflammation, time of flap creation, and enhance the ease of flap lifting [16].

In this study, there were few flap complications and most were mild. This may be due to the high frequency of the FS200 that permitted lower energy. Therefore, FS200 could reduce the incidence of flap complications when compared to a previous study [15]. For the incidence of Diffuse Lamellar Keratitis (DLK), one previous study found the rate of DLK after microkeratome LASIK was estimated to be 0.4% to 7.7%, whereas the rate after F-LASIK varied from 0.4% to 19.4% [17]. In this study, we found that the incidence of DLK was low (3.94%) and all were mild. There were no reports of the dysphotic phenomenon such as TLSS or rainbow glare in our case series, which may be underestimated due to the drawbacks of a retrospective study and it was also the limitation of this study.

In conclusion, the one-year clinical results of LASIK with the FS200 femtosecond laser and EX500 excimer laser showed high efficacy, predictability, stability and safety in 12 months. The results from the low to moderate myopic group were better than the high myopic group in terms of post-operative MRSE. However, there were no differences in UDVA at one year after surgery in both groups.

## Figures and Tables

**Fig. (1):**
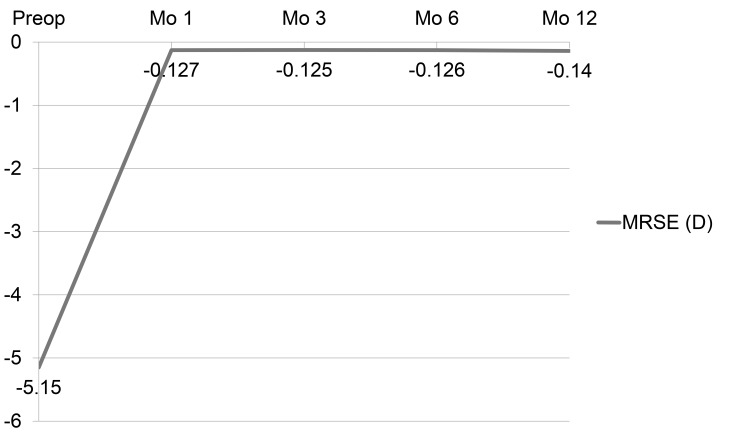
The changes of Mean Spherical Equivalent of Eyes that Underwent LASIK from Pre- to Post-operation at 1, 3, 6, and 12 months.

**Fig. (4):**
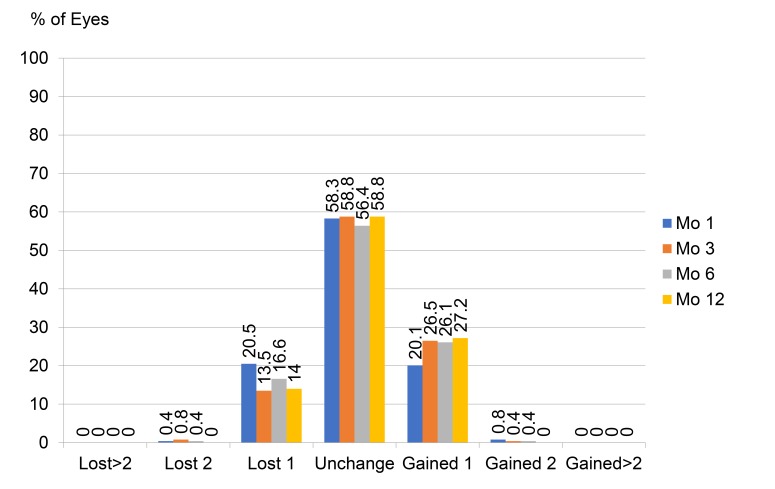
The change of best corrected distance visual acuity at 1, 3, 6, and 12 months post-surgery.

**Table 1:** Pre-operative data between low to moderate and high myopic group.

**Preoperative Data**	**Low to Moderate Myopia**	**High Myopia**	***P*-Value**
	**(N=161 eyes)**	**(N=93 eyes)**	
**N**o. of eyes (%)	161 (63.4%)	93 (36.6%)	–
Mean SE (D)	-3.60 ± 1.34	-7.83 ± 1.18	0.048
Mean UDVA (LogMAR)	0.91	1.3	<0.001
**Gender**	–	**–**	0.682
• Male	56 (34.8%)	30 (32.3%)	
• Female	105 (65.2%)	63 (67.7%)	
**Laterality**	–	**–**	0.578
• Right Eye	79 (49.1%)	49 (52.7%)	
• Left Eye	82 (50.9%)	44 (47.3%)	
Age (year)	32.32 ± 8.86	30.19 ± 8.03	0.424
CCT (µm)	544.17 ± 29.09	543.07 ± 25.11	0.215
Average K (D)	44.05 ± 1.28	44.08 ± 1.27	0.836

**Table 2:** Post-operative refractive outcomes compared between low to moderate and high myopic group.

**Postop data**	**1 month**			**3 months**			**6 months**			**12 months**		
	**Low**	**High**	***p*-value**	**Low**	**High**	***p*-value**	**Low**	**High**	***p*-value**	**Low**	**High**	***p-*value**
**Efficacy: UDVA**			0.017			0.014			0.061			0.091
• ≥20/10	10(6.2)	4(4.3)		11(7)	8(9.0)		14(9.3)	5(5.6)		13(8.2)	6(6.7)	
• ≥20/16	92(57.1)	34(36.6)		103(66)	42(47.2)		92(60.9)	39(43.3)		99(62.3)	41(45.6)	
• ≥20/20	145(90)	81(87.1)		141(90.4)	81(91.0)		141(93.4)	75(83.3)		147(92.5)	77(85.6)	
• ≥20/25	157(97.4)	89(95.7)		152(97.4)	86(96.6)		149(98.7)	86(95.5)		156(98.1)	84(93.3)	
• ≥20/32	158(98)	92(98.9)		154(98.7)	87(97.7)		151(100)	88(97.8)		157(98.7)	87(96.7)	
• ≥20/40	159(98.6)	92(98.9)		155(99.3)	86(97.7)		151(100)	89(98.9)		158(99.4)	88(97.8)	
**Predictability**			0.044			0.295			<0.001			0.006
• ±0.5 D	153(95)	82(88.2)		153(95)	85(92.4)		154(95.6)	78(86.7)		154(95.6)	77(83.7)	
• ±1.0 D	159(98.8)	91(97.8)		160(99.4)	90(97.8)		160(99.4)	88(97.8)		160(99.4)	89(96.7)	
• ±1.5 D	161(100)	92(98.9)		161(100)	90(97.8)		161(100)	89(98.9)		161(100)	91(98.9)	
• ±2.0 D	161(100)	93(100)		161(100)	92(100)		161(100)	90(100)		161(100)	92(100)	
**Stability**			0.001			0.007			<0.001			<0.001
• Mean SE (D)	-0.1	-0.18		-0.1	-0.17		-0.07	-0.23		-0.09	-0.23	
**Safety **			0.208			0.474			0.82			0.868
• Lost 2 lines	1(0.62)	0(0)		2(1.28)	0(0)		1(0.65)	0(0)		0(0)	0(0)	
• Lost 1 line	36(22.36)	16(17.2)		19(12.18)	15(16.67)		26(16.99)	15(16.48)		24(14.91)	12(12.9)	
• Unchanged	86(53.42)	62(66.7)		94(60.26)	50(55.56)		85(55.56)	52(57.14)		94(58.38)	54(58.06)	
• Gained 1 line	37(22.98)	14(15.1)		41(26.28)	24(26.67)		41(26.8)	23(25.27)		43(26.71)	27(29.03)	
• Gained 2 lines	1(0.62)	1(1.1)		0(0)	1(1.11)		0(0)	1(1.1)		0(0)	0(0)	

**Table 3:** Post-operative symptoms of dry eye, night vision problems, and corneal staining.

Eyes (%)	Post-operative Follow-Up Visit			
	1 Month	3 Months	6 Months	12 Months
**Dry Eye Symptoms Score**	–	–	–	–
• Grade 0 (none)	29 12.1)	42 (18.5)	68 (30.2)	72 (31.7)
• Grade 1-2	159 (66.2)	145 (63.9)	119 (52.9)	117 (51.5)
• Grade 3-5	52 (21.7)	40 (17.6)	38 (16.9)	38 (16.7)
• Average Score	1.68	1.45	1.28	1.19
**Night Vision Symptoms Score**	–	–	–	–
• Grade 0 (none)	55 (22.7)	94 (41.4)	103 (45.8)	124 (54.6)
• Grade 1-2	124 (51.2)	120 (52.9)	104 (46.3)	87 (38.4)
• Grade 3-5	63 (26.1)	13 (5.7)	18 (8.0)	16 (7.0)
• Average Score	1.58	0.89	0.82	0.71
**Signs**	–	–	–	–
Corneal Staining	101 (39.9)	57 (23.4)	35 (14.6)	13 (5.2)
• Mild	86 (33.9)	50 (20.6)	30 (12.6)	13 (5.2)
• Moderate	5 (2.0)	1 (0.4)	3 (1.2)	0 (0)
• Severe	10 (4.0)	6 (2.4)	2 (0.8)	0 (0)
